# Morphometric and hemodynamic parameter dataset for coronary artery aneurysms caused by atherosclerosis

**DOI:** 10.1016/j.dib.2019.104293

**Published:** 2019-07-19

**Authors:** Tingting Fan, Zhen Zhou, Wenxuan Fang, Wenjing Wang, Lei Xu, Yunlong Huo

**Affiliations:** a Department of Mechanics and Engineering Science, College of Engineering, Peking University, Beijing, China; b PKU-HKUST Shenzhen-Hongkong Institution, Shenzhen, China; c Department of Radiology, Beijing Anzhen Hospital, Capital Medical University, Beijing, China

**Keywords:** Aneurysm, Atherosclerosis, Morphometric, Hemodynamic

## Abstract

In comparison with intracranial aneurysm, there are relatively few investigations of coronary artery aneurysms (CAA). Coronary atherosclerosis is the first cause of CAA; therefore, it is necessary to providing as many details of clinical CAA caused by atherosclerosis as possible. The aim of the data is to provide morphometric and hemodynamic parameters of CAAs caused by atherosclerosis, as well as the demographics of patients with CAAs. Various morphometric parameters were obtained from the reconstructed epicardial coronary arterial trees of 61 patients while multiple hemodynamic parameters were determined from their computed flow fields. The data classified the CAAs into 4 types. All subjects in each group are listed in this data article. This data set support the main findings presented in the research article (Fan et al., 2019).

**Table:** Specifications Table

Subject area	*Biology*
More specific subject area	*Demographics, morphology, hemodynamics*
Type of data	*Tables*
How data was acquired	*Electrocardiogram(Hf-800b semi-automatic blood biochemical analyzer, HLIFE kangyu medical, jinan, China), Coronary CT (*256-row detector CT scanner [Revolution CT, GE Healthcare, Milwaukee, USA], 320–detector row [Aquilion One; Toshiba, Otawara, Japan], or dual-source [Somatom Definition Flash; Siemens, Forchheim, Germany] CT*), MIMICS (Materialise Company, Belgium), FLUENT (ANSYS Inc., Canonsburg, USA)*
Data format	*Raw, descriptive*
Experimental factors	*Age (year), Sex, Myocardial ischemia, Hypertension, Hyperlipidemia, Diabetes mellitus, Smoking, Systolic/Diastolic blood pressure (mmHg), Fasting glucose (mmol/L), Triglycerides (mmol/L), LDL (mmol/L), HDL (mmol/L), Total cholesterol (mmol/L), BMI (kg/m*^*2*^*), L/W (aneurysm shape index), Mean D*_*fit*_*of aneurysm (mm),*$L_{chord\;}/L_{arc}$*, Aneurysm sphericity (*φ*), SAR-TAWSS, SAR-OSI.*
Experimental features	*ST segment elevations as well as hyperacute T waves were used for determination of myocardial ischemia. Morphometric data were extracted based on CTA by MIMICS. Hemodynamic data are computed by FLUENT).*
Data source location	*Beijing Anzhen Hospital, Beijing, China; College of Engineering, Peking University, Beijing, China*
Data accessibility	*Data is attached with this article*
Related research article	*Fan T, Zhou Z, Fang W,* et al.*, Morphometry and hemodynamics of coronary artery aneurysms caused by atherosclerosis, Atherosclerosis, 2019;284;187–193*[1].

**Table:** 

**Value of the data** • This data could be a guideline for the study of functional morphology of CAAs as a result of coronary atherosclerosis. • This data could be helpful for the study of the morphometry and hemodynamics of patient-specific CAAs. Morphological and hemodynamic parameters are provided for 80 CAAs in 61 specific patients. • This data could be used in associated study of the morphology and hemodynamics of CAAs, clinical symptoms (i.e., myocardial ischemia, hypertension, hyperlipidemia) and patient info (i.e., age, gender, smoking and drinking history).

## 1 Data

The dataset presented in this article describes morphometric and hemodynamic parameters in epicardial coronary arteries of patients with CAAs caused by atherosclerosis. And it also provides the demographics of the CAA study population. There are 61 patients with 80 CAAs, which includes 10 CAAs of type I, 18 CAAs of type II, 29 CAAs of type III and 23 CAAs of type IV. Table 1 and Table 2 list the demographics (e.g., age, myocardial ischemia, diabetes mellitus) of 61 patients. Table 3 and Table 4 list the morphometric parameters (i.e., L/W, ${\text{L}}_{\text{chord}\;}/{\text{L}}_{\text{arc}}$, φ and Mean Dfit of aneurysm) for type I-IV CAAs. Table 5 and Table 6 list hemodynamic parameters (i.e., SAR-OSI and SAR-TAWSS) for type I-IV CAAs.

**Table 1:** Demographics of the type I and II CAA study population with CAAs (type IP1–P7 type II, P8–P18).

N.	Age (year)	Gender	MI	Hypertension	Hyperlipidemia	DM	Smoking	Systolic blood pressure (mmHg)	Systolic blood pressure (mmHg)	Fasting glucose (mmol/L)	TG (mmol/L)	LDL (mmol/L)	HDL (mmol/L)	TC (mmol/L)	BMI (kg/m^2^)
**L/W ≥ 2 and CAA covering a bifurcation**															
P1	69	M	Y	Y	Y	N	Y	139	96	5.6	2.00	3.01	1.01	5.09	32
P2	39	M	Y	Y	Y	N	Y	135	86	4.8	1.91	1.23	0.94	3.97	28.2
P3	62	M	N	Y	N	N	Y	135	95	4.2	1.05	2.3	0.96	3.32	26.5
P4	64	M	Y	Y	N	N	Y	116	70	5.6	1.02	1.36	1.02	3.277427	25.2
P5	40	M	N	N	Y	N	Y	110	68	5.3	2.31	2.46	1.04	3.17111	28.6
P6	63	M	Y	N	N	N	Y	110	60	5.8	1.02	1.72	0.95	2.63	26
P7	54	M	Y	N	N	N	Y	109	65	5.9	1.28	2.53	0.89	3.49	24
**L/W < 2 and CAA covering a bifurcation**															
P8	73	M	N	Y	N	N	Y	150	78	6.22	1.53	2.76	1.39	4.7	28.8
P9	73	M	Y	N	Y	N	Y	140	75	4.63	2.85	2.23	1.76	3.86	29.2
P10	70	M	N	N	Y	N	Y	115	78	5.41	2.75	2.72	1.42	3.84	27.1
P11	44	M	Y	Y	Y	N	Y	145	73	6.67	2.98	1.32	1.03	4.42	25.6
P12	52	M	N	Y	N	##	Y	136	74	6.37	1.64	1.56	0.82	3.82	29
P13	52	F	Y	Y	N	N	N	141	77	4.92	1.65	2.15	1.46	3.83	29.8
P14	52	F	N	Y	Y	N	N	132	75	5.01	2.01	1.93	1.6	4.15	24.5
P15	61	M	Y	N	N	N	N	120	73	4.77	1.65	2.27	0.97	3.78	24.9
P16	59	##	Y	N	N	##	N	132	77	6.97	1.55	1.84	1.94	4.4	25.5
P17	42	M	N	Y	N	Y	N	115	74	5.92	1.55	1.42	0.51	4.90	23.2
P18	43	##	Y	Y	Y	Y	N	132	78	7.53	2.45	2.49	1.01	4.85	20.3

**Table 2:** Demographics of the type III and IV CAA study population (type III, P19–P41l; type IV, P42–P461).

No.	Age (year)	Gender	MI	Hypertension	Hyperlipidemia	DM	Smoking	Systolic blood pressure (mmHg)	Systolic blood pressure (mmHg)	Fasting glucose (mmol/L)	TG (mmol/L)	LDL (mmol/L)	HDL (mmol/L)	TC (mmol/L)	BMI (kg/m^2^)
**L/W ≥ 2 and CAA in one vessel**															
P19	89	M	Y	Y	N	N	Y	130	80	5.76	0.63	1.86	1.05	3.2	34.3
P20	77	M	Y	Y	N	N	Y	135	86	4.23	0.95	1.91	0.95	3.17	31.2
P21	75	F	N	Y	N	N	N	144	88	5.51	1	2.32	0.85	3.61	32.5
P22	63	M	Y	Y	N	N	Y	135	86	4.63	0.95	1.91	0.95	3.17	31.5
P23	57	F	Y	Y	Y	N	N	125	83	4.86	2.97	2.64	0.79	4.4	31.0
P24	82	M	Y	Y	Y	N	Y	161	100	4.39	5.12	2.11	1.34	4.38	27.9
P25	53	M	N	N	Y	N	Y	150	92	4.70	3.01	2.53	0.89	4.63	19.0
P26	44	M	Y	Y	Y	N	Y	116	73	6.67	5.42	1.63	1.03	4.42	25.9
P27	31	M	N	N	Y	N	Y	144	92	5.13	5.57	3.71	1.76	6.22	27.5
P28	41	M	Y	N	N	N	N	137	79	4.77	0.71	2.27	0.97	3.6	25.6
P29	32	M	Y	N	N	N	N	83	53	5.84	0.44	2.15	1.29	3.64	17.0
P30	33	M	N	Y	Y	N	Y	148	87	5.3	2.44	2.84	1.04	5.03	28.7
P31	50	M	N	Y	Y	N	Y	124	79	6.37	2.39	3.75	1.07	7.07	22.4
P32	32	M	N	Y	Y	N	N	122	83	5.08	2.52	1.68	0.88	3.87	10.2
P33	61	M	Y	N	N	N	N	137	79	4.77	0.71	2.27	0.97	3.6	25.5
P34	35	M	N	Y	Y	N	N	120	80	4.68	3.32	1.91	0.73	3.51	20.7
P35	33	M	Y	N	N	N	N	83	53	5.84	0.44	2.15	1.29	3.64	19.4
P36	64	F	N	Y	N	N	N	119	70	5.40	0.79	1.67	0.94	3.20	26.3
P37	52	F	N	Y	N	N	N	118	73	6.36	0.93	1.86	0.85	4.60	32.6
P38	48	F	N	N	N	N	N	115	58	4.97	0.66	1.73	1.01	3.25	24.7
P39	46	F	N	N	N	N	N	105	79	4.86	0.16	1.90	1.24	3.65	16.3
P40	31	F	N	N	N	N	N	104	58	5.57	1.68	1.75	1.03	3.48	20.5
P41	50	##	N	N	N	N	N	88	72	5.86	1.67	1.80	0.99	3.67	21.6
**L/W < 2 and CAA in one vessel**															
P42	57	M	N	Y	Y	N	Y	125	75	6.22	1.31	2.76	1.39	4.7	31.4
P43	48	M	Y	N	Y	Y	N	120	73	15.02	13.58	2.28	0.89	7.41	19.1
P44	74	F	Y	Y	Y	Y	Y	135	100	14.29	13.37	3.39	0.96	6.38	28.4
P45	50	M	N	Y	Y	N	Y	148	95	6.37	2.39	3.86	1.07	7.07	28.2
P46	36	M	N	Y	Y	Y	Y	131	90	11.56	12.53	3.21	0.57	5.97	30.1
P47	59	M	Y	Y	N	N	Y	130	80	5.76	0.63	1.86	1.05	3.2	28.1
P48	62	M	N	Y	Y	N	N	128	74	5.3	2.06	3.62	1.01	5.36	27.9
P49	62	M	N	N	Y	##	Y	126	77	5.81	2.06	2.52	1.49	4.83	27.5
P50	68	F	Y	Y	Y	N	N	116	79	7.52	2.27	2.52	0.52	5.39	27.2
P51	52	F	N	Y	N	N	N	135	75	5.01	0.66	1.93	1.6	3.9	27.1
P52	54	M	Y	N	N	N	Y	138	95	6.65	1.28	2.39	1.17	3.92	26.8
P53	82	F	Y	Y	Y	N	N	133	85	8.31	1.53	3.10	1.16	3.12	25.9
P54	52	M	N	N	Y	N	N	101	59	5.41	2.56	3.37	0.9	5.74	25.8
P55	69	F	N	Y	N	N	N	101	67	5.47	2.87	2.37	1.06	4.32	25.1
P56	61	F	Y	Y	Y	N	Y	108	75	3.54	3.67	2.85	1.04	4.41	24.8
P57	52	M	Y	Y	N	N	Y	143	82	4.56	0.58	1.62	0.45	3.60	24.3
P58	61	M	N	N	Y	N	Y	116	68	5.23	2.34	2.36	1.13	4.42	24.0
P59	58	F	N	N	Y	N	N	129	73	7.19	0.66	2.02	1.23	5.33	22.8
P60	53	F	N	N	N	##	N	120	55	3.24	0.45	2.67	1.27	3.83	22.6
P61	40	F	N	N	N	##	N	119	55	3.74	0.36	2.19	1.11	2.52	22.5

**Table 3:** Morphometric parameters for type I and II CAAs (type I, C1–C10 and type II, C11–C28).

Aneurysm No.	Aneurysm shape index (L/W)	$\frac{L_{\mathrm{chord}\;}}{L_{\mathrm{arc}}}$	Mean D_fit_ of aneurysm (mm)	Aneurysm sphericity (ϕ)
**L/W ≥ 2 and CAAs covering a bifurcation**				
C1	3.1	0.9	4.6	0.8
C2	3.0	0.8	7.2	0.9
C3	3.0	0.8	7.9	0.8
C4	2.8	0.8	8.7	0.8
C5	2.7	0.7	5.4	0.8
C6	2.7	0.9	6.5	0.8
C7	2.5	0.8	7.3	0.9
C8	2.5	0.8	7.2	1.0
C9	2.4	0.9	7.0	0.9
C10	2.3	0.8	7.1	0.9
**L/W < 2 and CAAs covering a bifurcation**				
C11	1.8	0.9	6.7	0.9
C12	1.7	0.9	6.8	0.7
C13	1.6	0.9	4.2	1.1
C14	1.5	0.9	7.3	0.9
C15	1.4	0.8	7.4	1.2
C16	1.3	0.9	3.3	1.0
C17	1.3	0.8	3.4	0.9
C18	1.3	0.7	9.5	1.0
C19	1.3	0.9	6.2	1.0
C20	1.2	0.8	3.4	1.2
C21	1.2	0.9	4.0	1.3
C22	1.2	0.7	4.9	1.0
C23	1.1	0.8	2.7	0.4
C24	1.1	0.7	9.5	1.0
C25	1.1	0.7	3.2	1.0
C26	1.1	0.7	6.2	0.9
C27	1.1	0.7	0.8	0.1
C28	1.0	0.8	6.2	1.0

**Table 4:** Morphometric parameters for type III and IV CAAs (type III, C29–C57 and type IV, C58–C80).

Aneurysm No.	Aneurysm shape index (L/W)	$\frac{L_{\mathrm{chord}\;}}{L_{\mathrm{arc}}}$	Mean D_fit_ of aneurysm (mm)	Aneurysm sphericity (ϕ)
**L/W ≥ 2 and CAAs in one vessel**				
C29	5.2	0.6	10.2	0.6
C30	5.1	0.9	6.9	1.0
C31	5.1	0.7	7.9	0.7
C32	4.7	0.8	5.4	0.8
C33	4.7	0.4	6.6	0.7
C34	4.7	0.8	5.8	0.9
C35	4.3	0.6	6.4	0.8
C36	4.1	0.7	3.1	0.8
C37	4.0	0.8	6.8	0.8
C38	3.0	0.9	5.1	1.0
C39	2.9	0.8	4.0	0.9
C40	2.9	0.6	6.8	0.9
C41	2.9	0.9	4.5	0.9
C42	2.8	0.8	6.2	0.9
C43	2.8	0.6	4.4	0.9
C44	2.7	0.6	7.9	1.0
C45	2.7	0.9	5.0	0.8
C46	2.6	0.8	4.0	0.9
C47	2.6	1.0	3.2	0.9
C48	2.5	0.9	7.9	0.7
C49	2.5	0.7	3.3	1.0
C50	2.4	0.7	4.2	0.9
C51	2.4	0.4	5.6	0.8
C52	2.4	0.8	2.5	0.9
C53	2.3	0.9	7.9	0.8
C54	2.2	0.5	10.4	1.0
C55	2.2	0.8	4.3	0.8
C56	2.1	0.8	6.2	0.9
C57	2.0	0.8	7.1	0.7
**L/W < 2 and CAAs in one vessel**				
C58	1.9	0.5	6.8	1.2
C59	1.9	0.8	3.3	0.8
C60	1.9	1.0	4.9	1.1
C61	1.8	0.6	6.1	1.2
C62	1.8	1.1	6.4	1.0
C63	1.7	0.7	4.5	0.8
C64	1.6	0.7	4.7	1.0
C65	1.6	0.7	2.7	0.9
C66	1.6	0.7	7.9	0.9
C67	1.6	0.5	10.2	0.9
C68	1.6	0.9	4.5	1.0
C69	1.6	0.9	3.8	1.0
C70	1.5	0.8	3.7	1.1
C71	1.5	0.8	2.0	1.2
C72	1.4	0.9	5.0	0.8
C73	1.4	0.4	5.5	1.0
C74	1.3	0.8	2.2	1.1
C75	1.3	0.8	2.7	1.0
C76	1.3	0.4	4.9	0.9
C77	1.2	0.9	4.7	1.3
C78	1.2	0.9	7.5	1.0
C79	1.1	0.8	3.8	1.0
C80	1.1	0.9	3.8	1.0

**Table 5:** Hemodynamic parameters for type I and II CAAs (type I, C1–C10 and type II, C11–C28).

Aneurysm No.	SAR-OSI (%)	SAR-TAWSS (%)
**L/W ≥ 2 and CAAs covering a bifurcation**		
C1	13.6	47.5
C2	14.0	68.6
C3	11.8	29.0
C4	8.2	74.0
C5	6.0	39.8
C6	4.6	48.2
C7	0.9	36.3
C8	0.9	31.7
C9	7.0	40.6
C10	0.3	42.5
**L/W < 2 and CAAs covering a bifurcation**		
C11	0.4	18.8
C12	0.6	22.9
C13	0.5	22.1
C14	0.2	19.6
C15	0.6	27.1
C16	0.6	24.6
C17	0.3	20.8
C18	0.3	33.0
C19	0.1	31.9
C20	0.3	30.5
C21	0.6	27.5
C22	0.1	25.7
C23	0.1	16.5
C24	0.2	29.1
C25	0.3	25.4
C26	0.0	18.7
C27	0.1	17.0
C28	0.1	17.0

**Table 6:** Hemodynamic parameters for type III and IV CAAs (type III, C29–C57 and type IV, C58–C80).

Aneurysm No.	SAR-OSI (%)	SAR-TAWSS (%)
**L/W ≥ 2 and CAAs in one vessel**		
C29	18.4	49.0
C30	18.6	43.5
C31	15.0	39.3
C32	11.8	40.4
C33	17.2	44.8
C34	13.9	38.5
C35	9.0	20.3
C36	10.2	21.4
C37	8.8	35.8
C38	8.4	37.2
C39	7.1	13.5
C40	6.3	24.9
C41	4.2	35.2
C42	8.5	32.1
C43	4.0	27.4
C44	4.9	27.9
C45	3.5	32.0
C46	4.1	28.5
C47	4.5	32.7
C48	3.9	32.0
C49	1.1	26.0
C50	1.8	29.0
C51	1.4	20.3
C52	1.2	22.2
C53	2.7	22.7
C54	0.8	38.3
C55	1.1	18.2
C56	0.7	36.6
C57	1.6	21.0
**L/W < 2 and CAAs in one vessel**		
C58	1.1	14.1
C59	0.9	9.7
C60	0.5	14.3
C61	0.6	8.2
C62	0.3	19.4
C63	0.1	12.6
C64	0.4	9.5
C65	0.3	21.8
C66	0.1	6.4
C67	0.2	24.0
C68	0.1	20.4
C69	0.0	5.4
C70	0.1	27.8
C71	0.1	20.6
C72	0.0	16.1
C73	0.0	12.8
C74	0.1	1.5
C75	0.0	0.0
C76	0.1	4.9
C77	0.0	10.0
C78	0.1	0.4
C79	0.0	1.0
C80	0.1	2.0

## 2 Experimental design, materials and methods

### 2.1 Materials

The experiment shows the demographic data for 61 patients (patient numbers, P1–P61) with CAAs, who underwent coronary CT angiography (CTA) of the coronary arteries at the Beijing Anzhen Hospital, Beijing, China. A total of 80 coronary artery aneurysms (CAA number, C1–C80) were identified among these 61 specific patients. Multiple morphometric parameters are also defined. The study was approved by the Institutional Review Board (IRB) for the Beijing Anzhen Hospital, which conforms with the declaration of Helsinki.

### 2.2 Methods

Here, CAAs caused by atherosclerosis are divided into four groups in this data set. As the presence of a coronary artery bifurcation is the main major risk factor for CAAs followed by high aneurysm shape index (L/W, where L and W refer to the aneurysm length and maximum diameter, respectively); the characteristics of CAAs are grouped into type I (L/W ≥ 2 and CAA covering a bifurcation), type II (L/W < 2 and CAA covering a bifurcation), type III (L/W ≥ 2 and CAA in one vessel), and type IV (L/W < 2 and CAA in one vessel).

#### 2.2.1 Demographic data

General medical examinations, including medical history collection, blood pressure measurement, blood sampling, and urine analysis were performed. ST segment elevations as well as hyperacuity T waves were used for determination of myocardial ischemia. (Hf-800b semi-automatic blood biochemical analyzer, HLIFE kangyu medical, ji nan, China). Demographics of the study population, including age, sex, myocardial ischemia, hypertension, hyperlipidemia, diabetes mellitus, smoking, blood pressure, fasting blood glucose, triglycerides, cholesterol concentrations, and body mass index are listed in Table 1, Table 2.

1. LDL: low density lipoprotein
2. HDL: high density lipoprotein
3. BMI: body mass index
4. ##: unknown information
5. TC: total cholesterol
6. TG: triglycerides
7. MI: myocardial ischemia
8. DM: diabetes mellitus
9. DM: diabetes mellitus
10. Y: yes
11. N: no
12. M: male
13. F: female

#### 2.2.2 Morphometric data

Similar to previous studies [2], [3], the Coronary CTA was performed through three CT scanners (i.e., 256-row detector CT scanner [Revolution CT, GE Healthcare, Milwaukee, USA], 320–detector row [Aquilion One; Toshiba, Otawara, Japan], or dual-source [Somatom Definition Flash; Siemens, Forchheim, Germany] CT). All studies were of diagnostic image quality with optimal contrast enhancement and no substantial motion artifacts. All digitized data were imported into the MIMICS Innovation Suite platform (Materialise Company, Belgium) for 3D geometry reconstruction. Morphometric data of the epicardial coronary arteries with the CAA, i.e., L/W, ${\text{L}}_{\text{chord}\;}/{\text{L}}_{\text{arc}}$, φ and Mean Dfit of aneurysm were extracted based on the coronary CTA in each aneurysm (detailed definitions as follows).

1. L/W: aneurysm shape index, where W is maximum aneurysm diameter, L is aneurysm length.
2. φ: sphericity index=$\;\frac{{\text{π}}^{1/3}\cdot {\left(6\text{V}\right)}^{2/3}}{\text{A}}$, where V is the aneurysm volume and A is the surface area.
3. Mean D_fit_ of aneurysm (mm): the best fit diameter of the aneurysm, D_fit_, is calculated as twice the average radius between the point on the centerline and the contour of the 3D aneurysm vessel.
4. ${\text{L}}_{\text{chord}\;}/{\text{L}}_{\text{arc}}$: ${\text{L}}_{\text{chord}\;}$(mm) is the straight length from inlet to outlet of coronary artery and ${\text{L}}_{\text{arc}}$ (mm) is the accumulative length along the centerline of coronary artery.

The morphometric parameters for type I and II CAAs, which includes 10 type I CAAs and 18 type II CAAs, are listed in Table 3. The morphometric parameters for Type III and IV CAAs, which includes 29 type III CAAs and 23 type IV CAAs, are listed in Table 4.

#### 2.2.3 Hemodynamic data

Based on morphometric data, geometrical models were meshed using the ANSYS ICEM software (ANSYS Inc., Canonsburg, USA). The Navier-Stokes and continuity equations were solved using a finite volume solver, FLUENT (ANSYS Inc., Canonsburg, USA), as in previous studies [2], [3]. Three cardiac cycles were required to achieve convergence for the transient analysis. A constant time step was employed, where Δt = 0.01 s with 84 total time steps per cardiac cycle. The aortic pulsatile pressure wave was applied to the inlet of epicardial coronary arterial tree [2]. The resistance boundary condition was assigned to each outlet [2].

The time-averaged wall shear stress (TAWSS) and the oscillatory shear index (OSI) were obtained from the computed flow fields. From the data, we also computed SAR-TAWSS [4], [5] and SAR-OSI [6], [7] within the CAA region (detailed definitions as follows).

1. SAR-TAWSS within the CAA region: surface area ratio of low TAWSS ($=\frac{{\text{Surface}\;\text{area}}_{\text{TAWSS}\leq 4\;\text{dynes}\cdot {\text{cm}}^{-2}}}{\text{Aneurysmal}\;\text{surface}\;\text{area}}\times 100\text{\%}$) within the CAA region. Surface area of TAWSS ≤4 dyn/cm^2^ indicates the disease-prone site [4], [5].
2. SAR-OSI within the CAA region: surface area ratio of high OSI ($=\frac{{\text{Surface}\;\text{area}}_{\text{OSI}\geq 0.15}}{\text{Aneurysmal}\;\text{surface}\;\text{area}}\times 100\text{\%}$) within the CAA region. Surface area of OSI ≥0.15 indicates the disease-prone site [6], [7].

The hemodynamic parameters for type I and II CAAs, which includes 10 type I CAAs and 18 type II, are listed in Table 5. The hemodynamic parameters for type III and IV CAAs, which includes 29 type III CAAs and 23 type IV CAAs, are listed in Table 6.

## References

[1] Fan T., Zhou Z., Fang W. (2019). Morphometry and hemodynamics of coronary artery aneurysms caused by atherosclerosis. Atherosclerosis.

[2] Fan T., Feng Y., Feng F. (2017). A comparison of postoperative morphometric and hemodynamic changes between saphenous vein and left internal mammary artery grafts. Phys. Rep..

[3] Chen X., Gao Y., Lu B. (2016). Hemodynamics in coronary arterial tree of serial stenoses. PLoS One.

[4] Kleinstreuer C., Hyun S., Buchanan J.R. (2001). Hemodynamic parameters and early intimal thickening in branching blood vessels. Crit. Rev. Biomed. Eng..

[5] Malek A.M., Alper S.L., Izumo S. (1999). Hemodynamic shear stress and its role in atherosclerosis. J. Am. Med. Assoc..

[6] Huo Y., Luo T., Guccione J.M. (2013). Mild anastomotic stenosis in patient-specific CABG model may enhance graft patency: a new hypothesis. PLoS One.

[7] Nordgaard H., Swillens A., Nordhaug D. (2010). Impact of competitive flow on wall shear stress in coronary surgery: computational fluid dynamics of a LIMA-LAD model. Cardiovasc. Res..

